# Supplementary material for: Addressable and adaptable intercellular communication via DNA messaging
Source: Nat Commun. 2023 Apr 24;14:2358. doi: 10.1038/s41467-023-37788-z (PMC10126159; doi:10.1038/s41467-023-37788-z)
Supplement: Supplementary file 1 — Supplementary Information [file 41467_2023_37788_MOESM1_ESM.pdf]

Supplemental Information for: Addressable and adaptable intercellular communication via DNA messaging

John P. Marken[1*] and Richard M. Murray[1]

[1]Division of Biology and Biological Engineering, California Institute of Technology, Pasadena, CA
[*]To whom correspondence should be addressed: jmarken@caltech.edu

March 30, 2023

Supplementary Figures

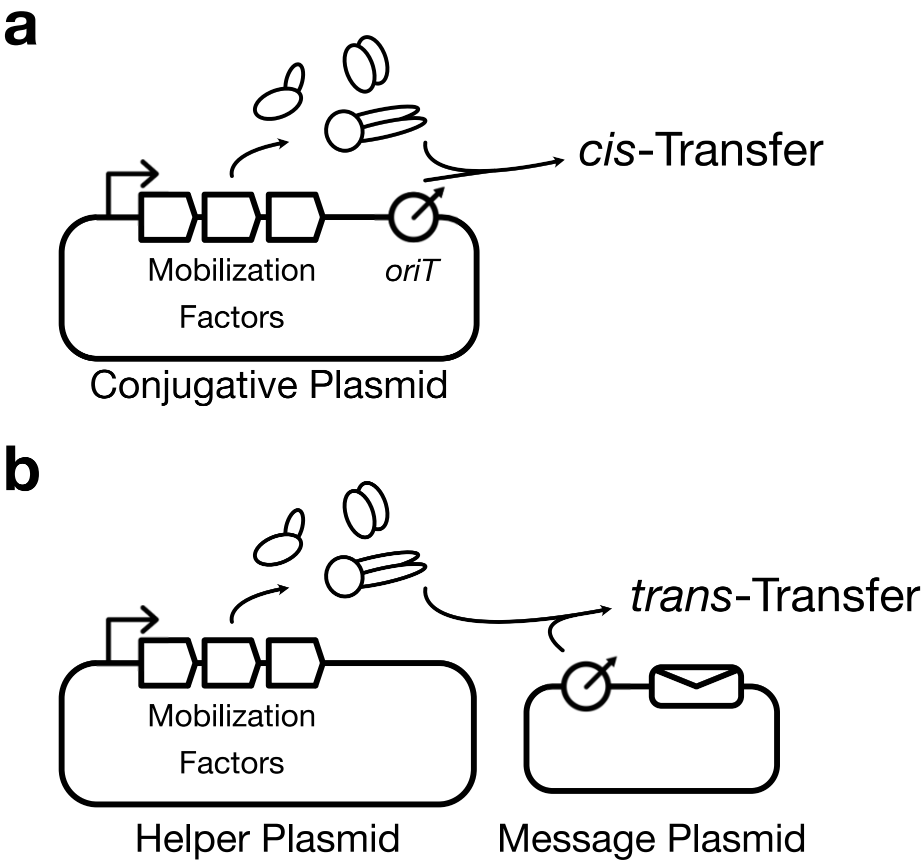

Supplementary Figure 1 How natural horizontal gene transfer systems are converted into DNA messaging channels. (a) Schematic of the architecture of a natural horizontal gene transfer system, using a conjugative plasmid as an example. The mobile vector expresses a set of genes, collectively called the mobilization factors, that transfer DNA elements that contain a cognate recognition sequence called the origin of transfer (*oriT*). Because the conjugative plasmid itself contains an *oriT* site, it transfers itself in a process termed *cis*-transfer. (b) Schematic of the architecture of a DNA messaging channel. The *oriT* is removed from the conjugative plasmid to create a helper plasmid that confers the ability to transfer DNA to its host cell but cannot transfer itself. The cognate *oriT* sequence can be placed onto another DNA vector to create a DNA message, which can then be transferred to another cell via the mobilization factors expressed by the helper plasmid. This process is called *trans*-transfer. Other horizontal gene transfer mechanisms, like non-lytic bacteriophages, share this same fundamental architecture and can be converted into DNA messaging channels through this same process.

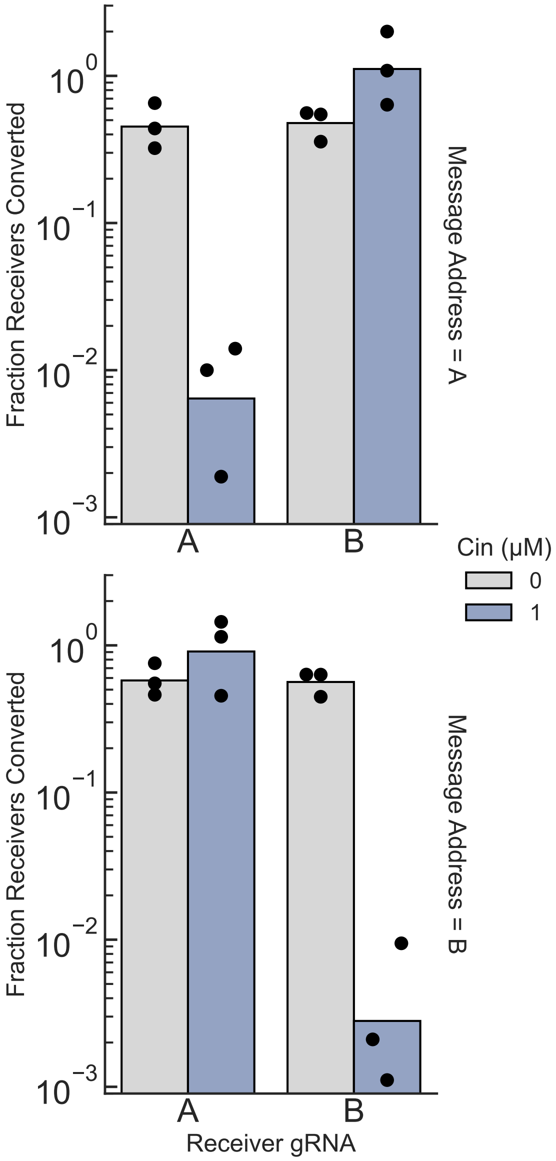

Supplementary Figure 2 Fractional receiver conversion of the experiments from Figure 3. Dots represent T/R values for each of three biological replicates measured on different days, calculated from the data shown in Figure 3b. Bars represent the geometric mean of the replicates. Source data are provided as a Source Data file.

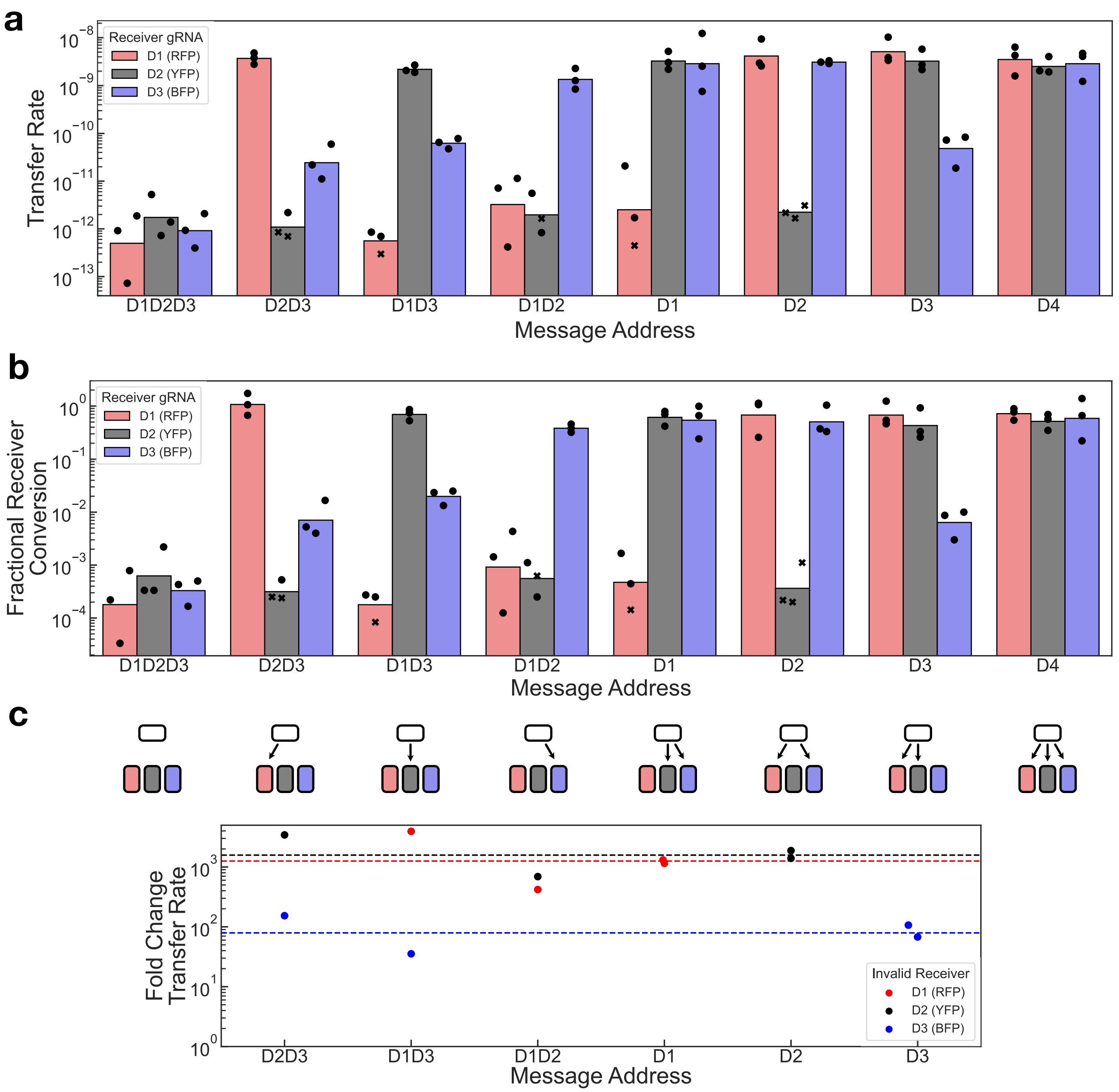

Supplementary Figure 3 Additional data from Figure 4. (a) Transfer rate values and (b) Fractional receiver conversion values from the experimental conditions shown in Figure 4b. Dots represent the values from each of three biological replicates measured on different days, and bars represent the geometric mean of these replicates. The value of the bars in (a) are identical to those plotted in the heatmap in Figure 4b. (c) Fold changes in the geometric means of the transfer rates between valid and invalid recipients for each message plasmid, based on the data in (a). For each message plasmid displayed on the horizontal axis, each dot represents one of the two possible choices of pairwise comparisons between a valid-receiver transfer rate and an invalid-receiver transfer rate. For the message plasmids with two binding sites, the single valid recipient is compared against the two possible invalid recipients, while for the message plasmids with one binding site, the two possible valid recipients are compared against the single invalid recipient. Each dot is colored by the identity of the invalid recipient in the comparison, and dashed lines represent the geometric mean across all of the fold change values where the recipient participated as the invalid comparison. Source data are provided as a Source Data file.

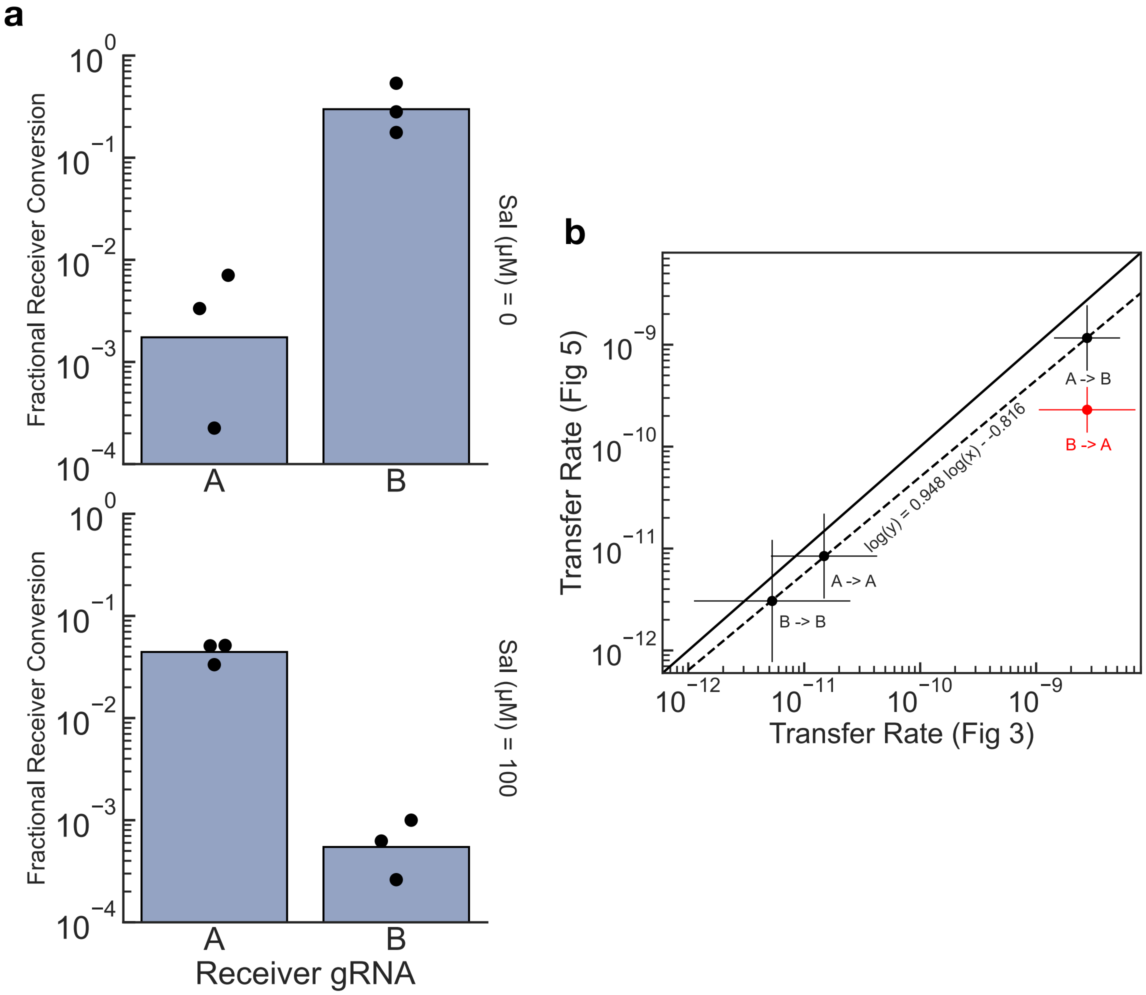

Supplementary Figure 4 Additional data from Figure 5. (a) Fractional receiver conversion values from Figure 5. Bars represent the geometric mean of the three biological replicates. (b) Plotting transfer rates from Figure 3 against those from Figure 5. Dots represent the geometric mean and error bars show one geometric standard deviation of the three biological replicates from each experiment. The solid line shows a direct log-linear relationship $\log(y) = \log(x)$, while the dotted line is the result of a log-linear fit to the three black points. The vertical distance between the solid line and each of the black points, indicating the global drop in transfer rate in the Figure 5 experiments, is (from left to right) 1.7, 1.8, and 2.4-fold (mean 2.0-fold). The vertical distance between the red point and the dotted line, indicating the additional drop in transfer rate for the post-edit on-target transfer, is 5.1-fold. Source data are provided as a Source Data file.

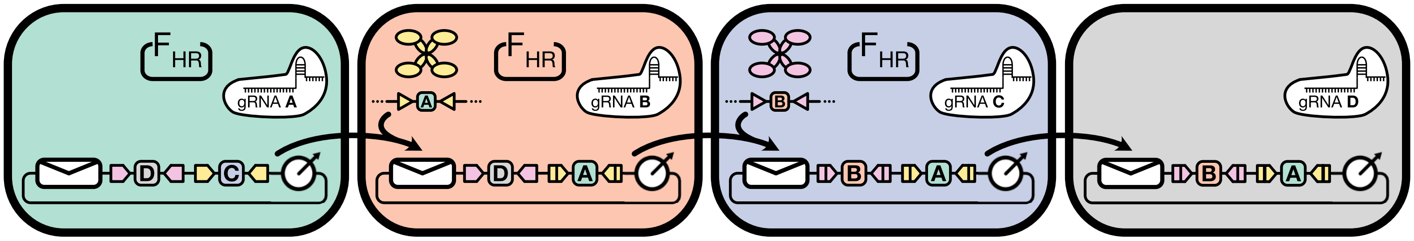

Supplementary Figure 5 Schematic of a four-strain linear relay. This architecture is preserved for any $n \geqslant 4$. Each strain in the population expresses one of n orthogonal gRNAs, and the address region on the message plasmid contains $n-2$ binding sites that block its transfer to all strains except its current strain and the next strain in the relay. Each site on the address is flanked by one of $n-2$ orthogonal integrase attachment site pairs. All strains except the last strain in the relay contain F_{HR} , and all strains except the first and last strains express a unique integrase that performs an address editing operation that invalidates the previous strain while validating the next strain in the sequence. Blocked transfers and intermediate message plasmid states are omitted from the diagram.

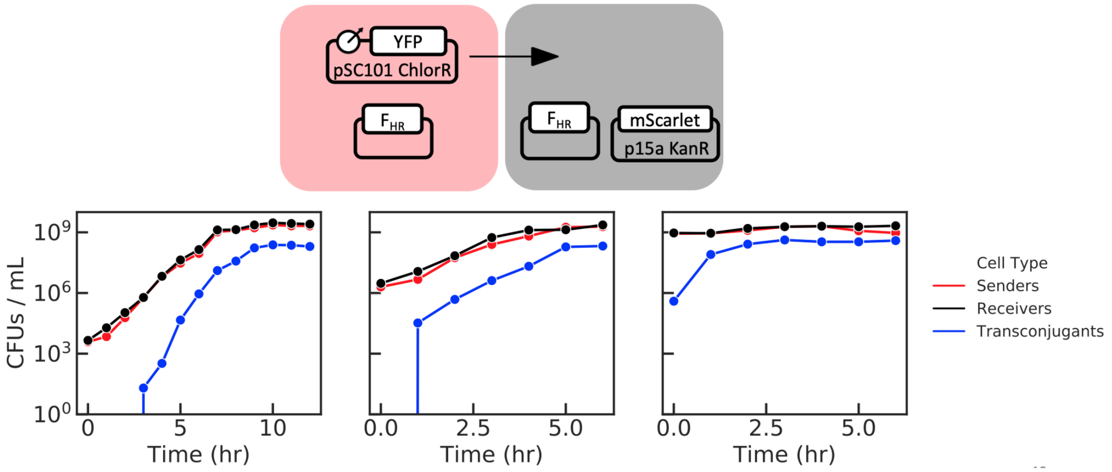

Supplementary Figure 6 Timecourse plating results of F_{HR} -mediated mating experiments without the transfer blocking system. Selective plating with chloramphenicol alone, kanamycin alone, or both antibiotics together was used to calculate the total sender, receiver, and transconjugant density, respectively. Experiments were performed in *E. coli* MG1655 cells, conducted in shaking LB media (as described in the Methods) without antibiotics. The three graphs represent three distinct biological replicates, each one having a different initial strain density for the coculture. In each case, the transconjugant density plateaus before overtaking the entire population. Source data are provided as a Source Data file.

Supplemental Note

One possible disadvantage of our Cas9-based addressing system is that genes on a message could transiently express within an invalid receiver cell in the window of time between the message's entry into the receiver and the message's degradation. In most cases, this transient expression phenomenon will likely not be problematic, as the invalid gene products will have a low concentration that is diluted out over time as the cells grow and divide (Supplementary Figure 7a). However, if these gene products are able to induce a longer-lasting change in the receiver cell's state during this transient window, then this phenomenon will become a major source of off-target transfers within the consortium (Supplementary Figure 7b).

We therefore set out to determine whether this transient expression phenomenon led to a detectable increase in off-target transfers under conditions similar to our experiments in the main text. In order to detect the presence of transient gene expression from a blocked message plasmid, we constructed receiver cells with a genomically-integrated cassette that can be edited by the BxbI integrase to permanently activate the chloramphenicol resistance gene. Because this activation occurs through a modification of the DNA sequence itself, this change is heritable. As such, the presence of chloramphenicol resistance in these receiver cells indicates that at one point its ancestry, the cell experienced sufficiently high BxbI expression to activate the resistance cassette. A lack of chloramphenicol resistance in the receivers, therefore, can be used as stringent evidence for the inability of BxbI to accumulate in receiver cells to functional levels.

We constructed our message plasmids to express destabilized BxbI in the receiver cells by placing them under a salicylate-inducible promoter whose cognate transcription factor is only expressed in the receiver strain. We constructed two variants of these BxbI message plasmids that differ only in their ribosome binding site, so that one plasmid expresses BxbI weakly while the other expresses it strongly. We used the high-copy ColE1 origin of replication for these message plasmids, unlike the low-copy pSC101 origin used in the experiments in the main text, to ensure that the weakly-expressing BxbI plasmid could still produce a sufficient amount of BxbI to activate the chloramphenicol resistance cassette in the receivers (Supplementary Figure 7c).

We then conducted pairwise sender-receiver experiments where we transferred these BxbI message plasmids, bearing the A address site, to receiver strains expressing either the A or B gRNA. In order to measure our system's baseline ability to block ColE1 message plasmids, we constructed another message plasmid variant that expresses YFP and the chloramphenicol resistance gene directly (Supplementary Figure 7c). Because transconjugants were selected with chloramphenicol in all conditions, the YFP plasmid transfer rate captures only transconjugants that currently contain the message plasmid while the BxbI plasmid transfer rate captures receiver cells that at one point in their ancestry received the plasmid for sufficient time to express BxbI and activate the chloramphenicol resistance cassette.

All three message plasmids transferred successfully to the B gRNA receiver, indicating that the BxbI message plasmid, even when weakly expressing the integrase, is able to activate the chloramphenicol resistance gene in the receivers (Supplementary Figure 7d).

We next calculated the effectiveness of the gRNA system at blocking the message plasmids by calculating what we term the Block Fidelity, which is the observed transfer rate to the valid recipient (B gRNA) divided by the transfer rate to the invalid recipient (A gRNA) (Supplementary Figure 7e). A higher value of Block Fidelity indicates more effective blocking of the invalid message plasmid. By normalizing the Block Fidelity of each BxbI plasmid by the Block Fidelity of the YFP plasmid, we obtained a value that we call the Transient Expression Index (Supplementary Figure 7f). If our assay was unable to detect any transient expression of BxbI in the invalid receiver cells, then the Transient Expression Index should take a value of 1. Values greater than 1 indicate that transient expression was detected by our assay.

We observed that the weakly-expressing BxbI plasmid had a Transient Expression Index near 1, while the strongly-expressing BxbI plasmid had a Transient Expression index near 10 (Supplementary Figure 7f). This result indicates that the weakly-expressing BxbI message plasmid was not able to transiently express enough BxbI to activate the chloramphenicol resistance gene in the receivers, while the strongly-expressing message plasmid could. We can therefore conclude that it is possible to create message plasmids for which the transient expression of their encoded genes within an invalid receiver cell will not be a significant factor.

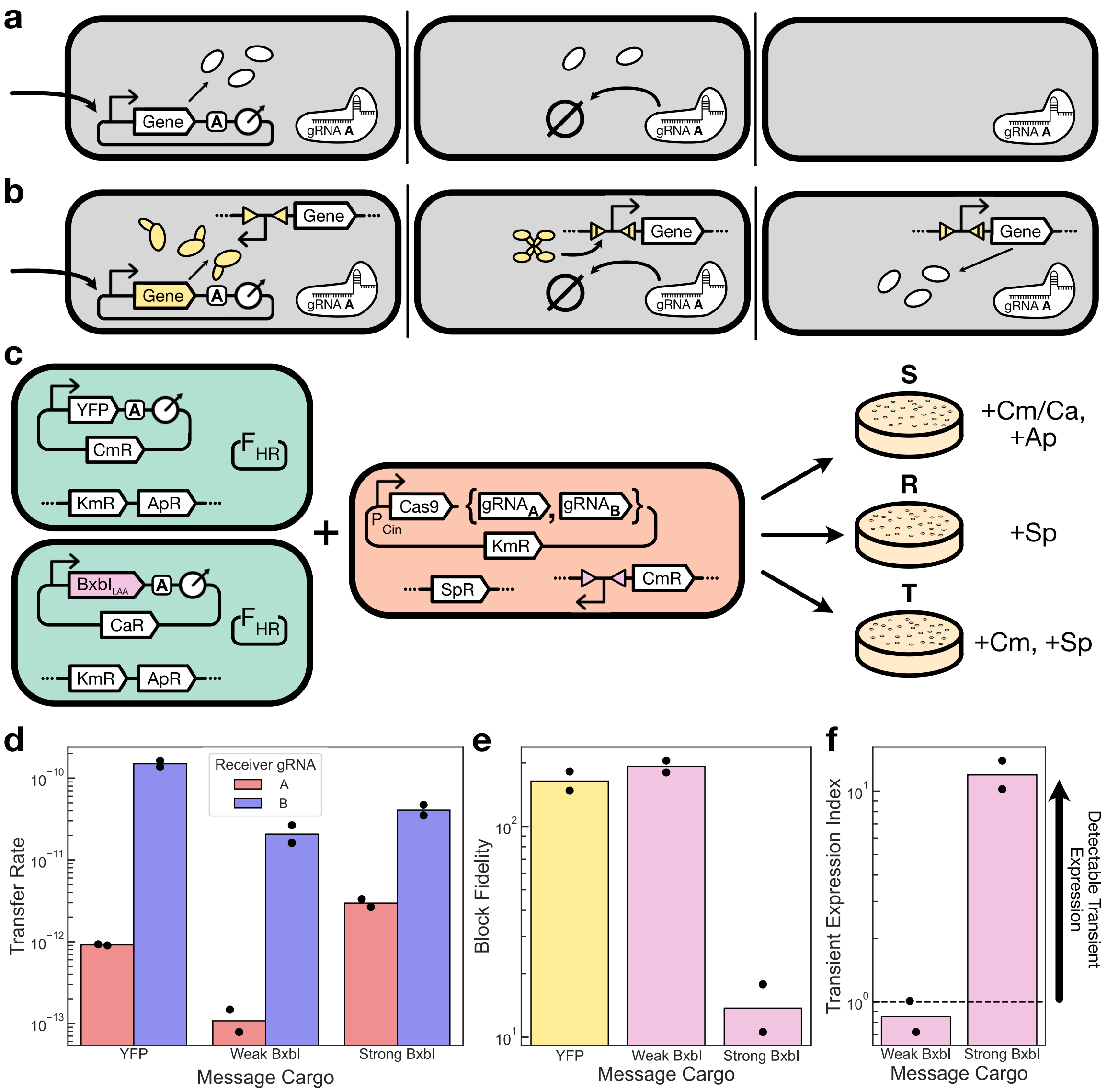

Supplementary Figure 7 Transient Expression from blocked messages. (a) Schematic of transient expression from a blocked message. (Left panel) An invalid message enters the recipient cell and begins expressing genes. (Middle panel) The Cas9-gRNA complex degrades the message plasmid, but the expressed proteins remain in the cell. (Right panel) Eventually, these proteins are degraded or diluted out of the cell and the influence of the invalid message disappears. (b) Schematic of transient expression inducing a permanent change in the receiver cell. The fate of the plasmid is identical to (a), but its cargo catalyzes a permanent genetic change in the receiver that persists after the plasmid's removal. (c) Experimental schematic. Two types of message plasmids were constructed: one carrying YFP and chloramphenicol resistance cargo, and one carrying a degradation-tagged BxbI integrase and carbenicillin resistance cargo. These were mixed in pairwise transfer experiments with receiver strains expressing a cognate or non-cognate gRNA and a BxbI-activatable chloramphenicol resistance cassette integrated onto the genome. Transconjugants were always selected with chloramphenicol, so that the YFP plasmid transfer rate captures only transconjugants that currently contain the message plasmid while the BxbI plasmid transfer rate captures receiver cells that at one point in their ancestry received the plasmid for sufficient time to express a functional level of BxbI. (d) Measured transfer rates for the YFP and BxbI plasmids. Two variants of the BxbI plasmids, identical except for their expression strength, were tested. (e) The block fidelity, defined as the ratio of the transfer rates between the valid and invalid receiver, for the experiments. Higher values indicate better blocking. (f) The transient expression index, defined as the ratio of block fidelity between the BxbI plasmid and the YFP plasmid. A value > 1 indicates that the YFP plasmid was blocked more effectively than the BxbI plasmid, indicating that transient expression of the BxbI in invalid receiver cells occurred. A value $\leqslant 1$ indicates that transient expression of BxbI in the invalid receiver cells was not detectable. Dots represent two biological replicates measured on different days, and bars indicate their geometric mean. Km = kanamycin, Cm = chloramphenicol, Ca = carbenicillin, Ap = apramycin, Sp = spectinomycin. Source data are provided as a Source Data file.
