# Supplementary material for: Addressable and adaptable intercellular communication via DNA messaging
Source: Nat Commun. 2023 Apr 24;14:2358. doi: 10.1038/s41467-023-37788-z (PMC10126159; doi:10.1038/s41467-023-37788-z)
Supplement: Supplementary file 2 — Reporting Summary [file 41467_2023_37788_MOESM2_ESM.pdf]

nature portfolio

Corresponding author(s): John P. Marken

Last updated by author(s): Mar 13, 2023

Reporting Summary

Nature Portfolio wishes to improve the reproducibility of the work that we publish. This form provides structure for consistency and transparency in reporting. For further information on Nature Portfolio policies, see our Editorial Policies and the Editorial Policy Checklist.

Statistics

For all statistical analyses, confirm that the following items are present in the figure legend, table legend, main text, or Methods section.

n/a	Confirmed	
☐	☒	The exact sample size (*n*) for each experimental group/condition, given as a discrete number and unit of measurement
☐	☒	A statement on whether measurements were taken from distinct samples or whether the same sample was measured repeatedly
☒	☐	The statistical test(s) used AND whether they are one- or two-sided<br>*Only common tests should be described solely by name; describe more complex techniques in the Methods section.*
☒	☐	A description of all covariates tested
☒	☐	A description of any assumptions or corrections, such as tests of normality and adjustment for multiple comparisons
☐	☒	A full description of the statistical parameters including central tendency (e.g. means) or other basic estimates (e.g. regression coefficient) AND variation (e.g. standard deviation) or associated estimates of uncertainty (e.g. confidence intervals)
☒	☐	For null hypothesis testing, the test statistic (e.g. *F*, *t*, *r*) with confidence intervals, effect sizes, degrees of freedom and *P* value noted<br>*Give P values as exact values whenever suitable.*
☒	☐	For Bayesian analysis, information on the choice of priors and Markov chain Monte Carlo settings
☒	☐	For hierarchical and complex designs, identification of the appropriate level for tests and full reporting of outcomes
☒	☐	Estimates of effect sizes (e.g. Cohen's *d*, Pearson's *r*), indicating how they were calculated

Our web collection on statistics for biologists contains articles on many of the points above.

Software and code

Policy information about availability of computer code

Data collection	MicroManager version 2.0.0 was used to operate the Olympus MVX10 microscope to capture images of the plates.
Data analysis	CFU counts were obtained manually by viewing the plate images in ImageJ version 1.51s or directly by eye. Analyses of CFU counts (calculation of geometric means and fold changes) were performed using custom Python scripts written for Python 3.8.8 that are available on GitHub at github.com/jpmarken/DNAmessaging.<br><br>The A and B gRNA sequences were compared against the E. coli genome (taxid:562) using BLASTN 2.13.0+ to confirm a lack of homology.

For manuscripts utilizing custom algorithms or software that are central to the research but not yet described in published literature, software must be made available to editors and reviewers. We strongly encourage code deposition in a community repository (e.g. GitHub). See the Nature Portfolio guidelines for submitting code & software for further information.

Data

Policy information about availability of data

All manuscripts must include a data availability statement. This statement should provide the following information, where applicable:

- Accession codes, unique identifiers, or web links for publicly available datasets
- A description of any restrictions on data availability
- For clinical datasets or third party data, please ensure that the statement adheres to our policy

Colony count data for each experiment is provided in the Source Data.

A description of all of the strains and constructs created for this study, as well as the GenBank accession IDs that provide their sequences, can be found in Supplemental Data 1 but are also reproduced here below.

Plasmids:
OQ603057
OQ603058
OQ603059
OQ603060
OQ603061
OQ603062
OQ603063
OQ603064
OQ603065
OQ603066
OQ603067
OQ603068
OQ603069
OQ603070
OQ603071
OQ603072
OQ603073
OQ603074
OQ603075
OQ603076

Genomic cassettes:
OQ603077
OQ603078
OQ603079
OQ603080
OQ603081
OQ603082
OQ603083

Human research participants

Policy information about studies involving human research participants and Sex and Gender in Research.

Reporting on sex and gender	N/A
Population characteristics	N/A
Recruitment	N/A
Ethics oversight	N/A

Note that full information on the approval of the study protocol must also be provided in the manuscript.

Field-specific reporting

Please select the one below that is the best fit for your research. If you are not sure, read the appropriate sections before making your selection.

- [x] Life sciences
- [ ] Behavioural & social sciences
- [ ] Ecological, evolutionary & environmental sciences

For a reference copy of the document with all sections, see nature.com/documents/nr-reporting-summary-flat.pdf

Life sciences study design

All studies must disclose on these points even when the disclosure is negative.

Sample size	Unless otherwise indicated, experiments were performed across three biological replicates measured on different days, following standard procedure in the field (see e.g. Meyer et al 2019, Nature Chemical Biology). No statistical method was used to predetermine an appropriate sample size for any of the experiments. The following experiments were performed with fewer replicates: Fig S7 was performed with two biological replicates and the timecourse experiments (Figs 6c, S6) were performed with one biological replicate. The timecourse experiments were not repeated additional times due to their laborious nature, and the Fig S7 experiment was not repeated additional times because the two replicates were sufficient to support the conclusion drawn from the results, i.e. that it is possible for the Transient Expression Index to take a value of ~1 in at least one experimentally-accessible condition.
Data exclusions	Data were only excluded from the analyses when an obvious experimental error was detected, for example when the strain labels on the selection plates were not consistent with the antibiotic profiles of the plates themselves. In such conditions the entire dataset from that day was thrown out, including other conditions where an obvious error was not necessarily detectable, and the experiment was repeated on a different day.
Replication	Experiments were performed in biological triplicate on different days (with the exception of the cases noted in 'Sample Size' above) in order to capture both biological and technical variability within the results, and in general the salient takeaway conclusions from the experiments are qualitative rather than quantitative (i.e. it is more important that the transfer rate was detectably biased towards the desired recipient, rather than the fact that it was biased to an X-fold degree). For the most complex experiment, the linear relay in Fig 6, the performance of the system was assessed by three distinct approaches (endpoint strain densities, strain growth dynamics, and PCR assays of the message plasmids) to support the reliability of their conclusions. Results were consistent between replicates.
Randomization	For each experiment, each biological replicate was sourced from a single randomly-selected colony from a streak of a stock of the appropriate strain grown overnight. This colony was then propagated in liquid media and used that day for every experimental condition within the experiment.
Blinding	Investigators were not blinded to the grouping of the samples, which was necessary in order to ensure the proper setup of the experiments via the addition of the appropriate strains, antibiotics, and inducers to the various cultures.

Reporting for specific materials, systems and methods

We require information from authors about some types of materials, experimental systems and methods used in many studies. Here, indicate whether each material, system or method listed is relevant to your study. If you are not sure if a list item applies to your research, read the appropriate section before selecting a response.

Materials & experimental systems

n/a	Involved in the study	
☒	☐	Antibodies
☒	☐	Eukaryotic cell lines
☒	☐	Palaeontology and archaeology
☒	☐	Animals and other organisms
☒	☐	Clinical data
☒	☐	Dual use research of concern

Methods

n/a	Involved in the study	
☒	☐	ChIP-seq
☒	☐	Flow cytometry
☒	☐	MRI-based neuroimaging
