# Supplementary material for: Addressable and adaptable intercellular communication via DNA messaging
Source: Nat Commun. 2023 Apr 24;14:2358. doi: 10.1038/s41467-023-37788-z (PMC10126159; doi:10.1038/s41467-023-37788-z)
Supplement: Supplementary file 3 — Description of Additional Supplementary Files [file 41467_2023_37788_MOESM3_ESM.pdf]

Title: Supplementary Data 1

Description: A description of the strains and constructs created for this study and the figures in which they are used. Also includes Genbank accession IDs for the annotated sequences of all new constructs.
